# Functionalized nanoparticles with monocyte membranes and rapamycin achieve synergistic chemoimmunotherapy for reperfusion-induced injury in ischemic stroke

**DOI:** 10.1186/s12951-021-01067-0

**Published:** 2021-10-21

**Authors:** Yanyun Wang, Yi Wang, Shuyu Li, Yuliang Cui, Xiping Liang, Juanjuan Shan, Wei Gu, Juhui Qiu, Yiliang Li, Guixue Wang

**Affiliations:** 1 Key Laboratory for Biorheological Science and Technology of Ministry of Education, State and Local Joint Engineering Laboratory for Vascular Implants, Bioengineering College of Chongqing University, Chongqing, 400030 China; 2 Department of Hematology-Oncology, Chongqing Key Laboratory of Translational Research for Cancer Metastasis and Individualized Treatment, Chongqing University Cancer Hospital, Chongqing, 400030 China; 3 Center for Precision Medicine of Cancer, Chongqing Key Laboratory of Translational Research for Cancer Metastasis and Individualized Treatment, Chongqing University Cancer Hospital, Chongqing, China; 4 The Eighth Affiliated Hospital, Sun Yat-Sen University, Shenzhen, 518033 Guangdong China

**Keywords:** Stroke, Ischemia/reperfusion injury, Monocyte membrane, Rapamycin, Nanoparticles

## Abstract

**Background:**

Ischemic stroke is an acute and severe neurological disease, and reperfusion is an effective way to reverse brain damage after stroke. However, reperfusion causes secondary tissue damage induced by inflammatory responses, called ischemia/reperfusion (I/R) injury. Current therapeutic strategies that control inflammation to treat I/R are less than satisfactory.

**Results:**

We report a kind of shield and sword nano-soldier functionalized nanoparticles (monocyte membranes-coated rapamycin nanoparticles, McM/RNPs) that can reduce inflammation and relieve I/R injury by blocking monocyte infiltration and inhibiting microglia proliferation. The fabricated McM/RNPs can actively target and bind to inflammatory endothelial cells, which inhibit the adhesion of monocytes to the endothelium, thus acting as a shield. Subsequently, McM/RNPs can penetrate the endothelium to reach the injury site, similar to a sword, and release the RAP drug to inhibit the proliferation of inflammatory cells. In a rat I/R injury model, McM/RNPs exhibited improved active homing to I/R injury areas and greatly ameliorated neuroscores and infarct volume. Importantly, in vivo animal studies revealed good safety for McM/RNPs treatment.

**Conclusion:**

The results demonstrated that the developed McM/RNPs may serve as an effective and safe nanovehicles for I/R injury therapy.

**Graphic abstract:**

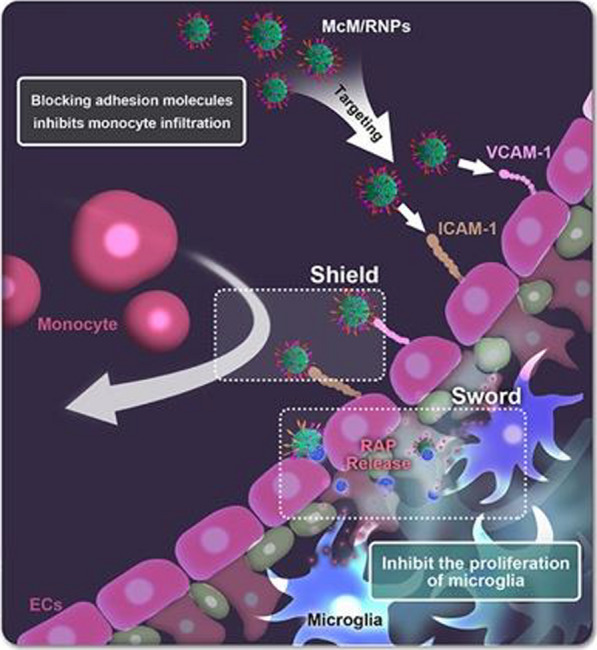

**Supplementary Information:**

The online version contains supplementary material available at 10.1186/s12951-021-01067-0.

## Background

Despite recent therapeutic advances, stroke remains the leading cause of disability with high social and economic burden [1]. Ischemic stroke represents ~ 71% of all strokes worldwide and is associated with infarction of the brain, spinal cord, or retina [2]. Reperfusion for cerebral ischemia is an effective approach to reducing disability when administered within 4.5 h after stroke [3]. However, cerebral ischemia/reperfusion (I/R) can also lead to serious injury to the brain, neural cells, and even the entire body because of the abrupt recovery of blood flow and strong inflammation [3]. Accordingly, there is an urgent need for innovative and effective therapeutic approaches for cerebral I/R.

With intensive study of post ischemic microenvironment, apoptotic and dead neuronal cells release cellular factors, and cytokines induce strong inflammation in the injured brain region [4]. A growing body of evidence suggests that inflammatory responses persist throughout the entire brain for a long time and affect patients' long-term neurological outcomes. In addition, microglia in the focal injury site can activate infiltrating macrophages and other inflammatory cells [5, 6]. Therefore, deletion of chemokines, such as mineralocorticoid receptor and transforming growth factor beta-activated kinase 1, can alter inflammation and reduce infarct volume during cerebral ischemia [7]. Furthermore, focal brain inflammation aggravates secondary brain injury by exacerbating blood–brain barrier damage and microvascular failure [4]. The permeability of endothelial cells is also critical for the blood–brain barrier (BBB) and inflammation in the ischemia–reperfusion brain, with a vicious circle [8]. Overall, reducing local microglial activation and inflammation through endothelial cells is a potential way to control the inflammation after cerebral I/R.

With the rapid development of nanotechnology, nanoparticles with different components have recently developed and can efficiently reverse reperfusion-induced injury in ischemic stroke. Most of the mechanisms are nanoparticles with prolonged blood circulation time, reduced clearance rate, improved BBB penetration ability, and enhanced brain accumulation [9]. Reducing neuroinflammation has become an effective adjunct therapy to improve outcomes [10]. There are two main approaches to abate inflammation: reducing the inflammatory cells penetrating into the injury site from the blood and inhibiting inflammatory cell activation and proliferation in the focal site. The BBB and inflammatory endothelial cells are critical for both drug delivery and inflammatory cell infiltrating in stroke therapies [11]. Neutrophil-derived nanovesicles can alleviate inflammation to protect against ischemic stroke injury by decreasing adhesion neutrophils with endothelial cells [12]. To decrease inflammation, protein nanoparticle-conjugated doxorubicin (DOX) can alleviate neurological damage in stroke by inducing neutrophil apoptosis [13]. Nanoparticles can mediate siRNA delivery to protect neurons from cerebral I/R by inhibiting microglial neurotoxicity [14]. Nanocurcumin was also found can protect the blood–brain barrier and reduce M1 microglial activation simultaneously [15].

To inhibit the continuous recruitment of monocyte, monocyte cell membrane nanoparticles can directly bind with inflammatory endothelial cells to cut off the critical pathway for monocyte entry into the injury site [12]. In fact, our previous research showed that nanoparticles from the macrophage cell membrane can inhibit atherosclerosis development [15]. For the microglia already existing in the brain, it is imperative to weaken their function and proliferation in the original site. For example, rapamycin (RAP) can significantly decrease the production of proinflammatory cytokines and chemokines by macrophages and microglia [16, 17].

**Scheme 1.**
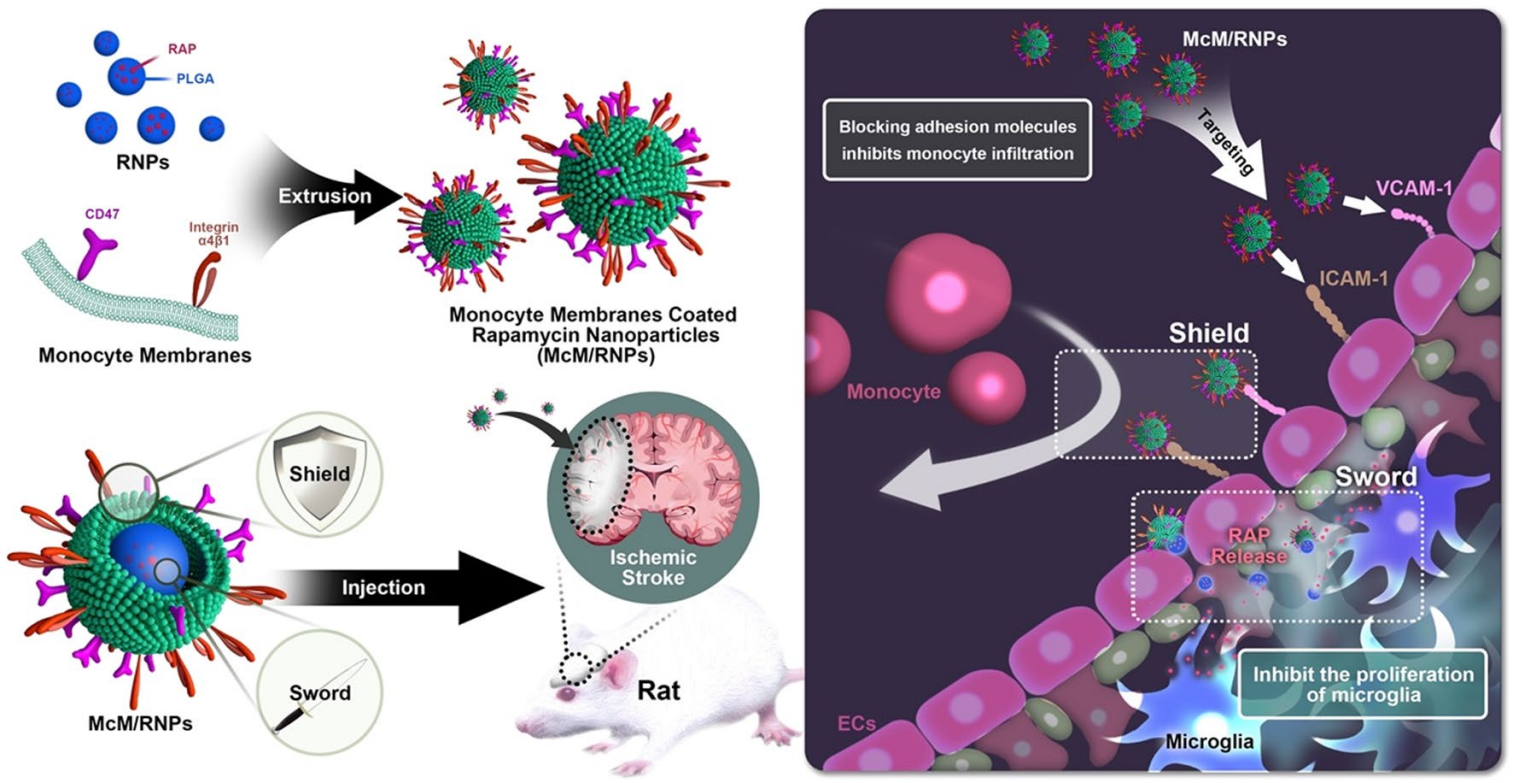
Schematic of McM/RNPs fabrication and its treatment for I/R

Here, we introduce shield and sword nano-soldiers functionalized nanoparticles with monocyte membrane (McM) and rapamycin (RAP) to protect against reperfusion-induced injury in ischemic stroke. This nanoparticle was fabricated by coating the membrane derived from monocytes onto preformed poly (lactic-co-glycolic acid) (PLGA) cores loaded with RAP, named McM/RNPs, to achieve synergistic immuno-chemotherapy. McM/RNPs can actively bind to inflammatory endothelial cells, which act as a shield to resist the recruitment of inflammatory cells to the brain, thereby cutting off the ‘fuel supply’ for uncontrollable inflammation. Subsequently, McM/RNPs penetrate the endothelium to reach the injury site and release the RAP drug, which acts as a sword to relieve inflammation by inhibiting the proliferation of microglia in the injury site. This design of nanoparticles provides a new strategy to inhibit neuroinflammation with immunotherapy and enhance the neural regeneration of tMCAO rats (Scheme 1).

## Results and discussion

### Assembly and characterization of McM/RNPs

**Fig. 1:**
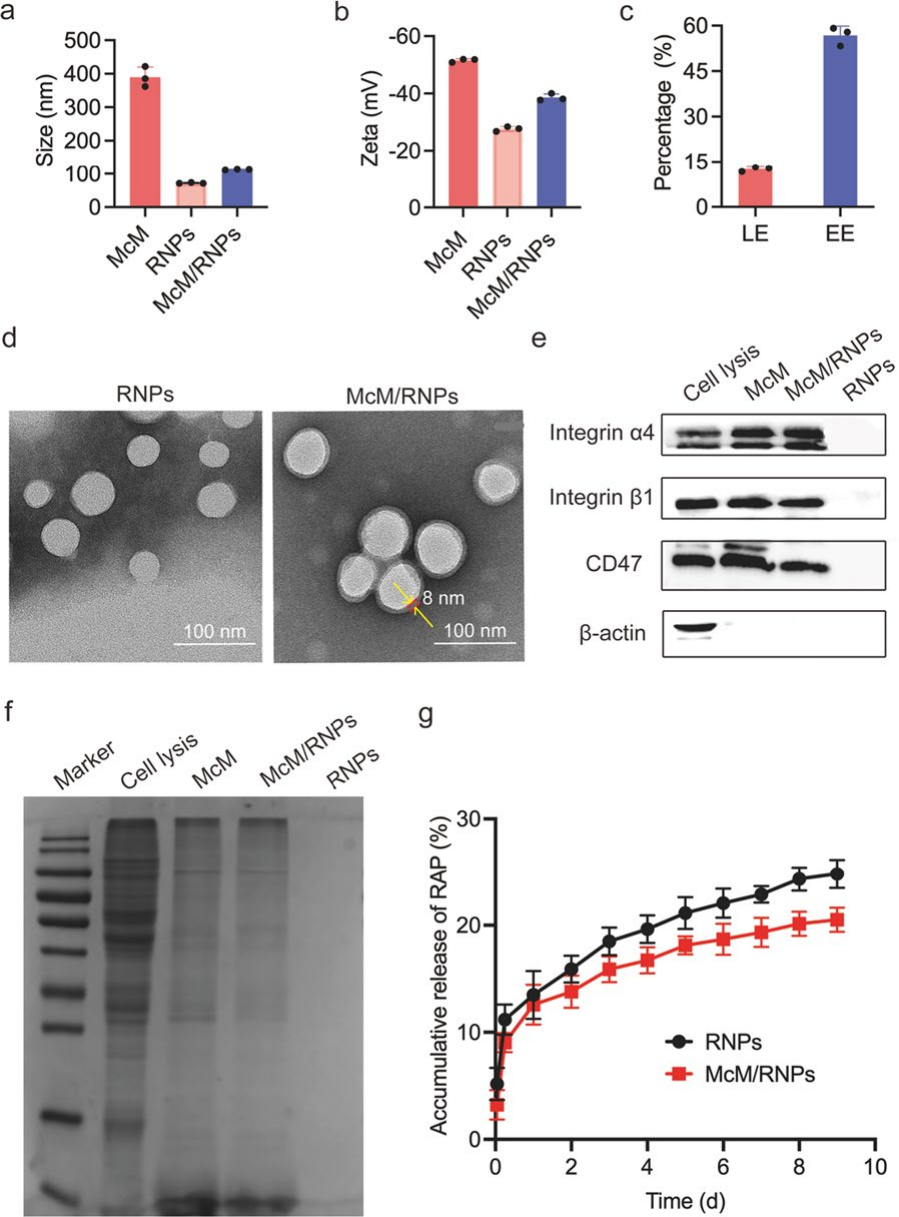
Characterization of the McM/RNPs. **a** The sizes and **b** zeta potentials of McM, RNPs and McM/RNPs (*n* = 3, mean ± SD). **c** Drug loading efficiency (LE) and encapsulating efficiency (EE) of RNPs. **d** TEM images of RNPs and McM/RNPs (scale bar = 100 nm). **e** Western blotting results of integrin α4 (150 kDa /140 kDa), integrin β1 (135 kDa), CD 47 (35 kDa) and β-actin (42 kDa). **f** Proteins in cell lysis, McM, McM/RNPs, and RNPs by SDS-PAGE. Coomassie brilliant blue staining was used to detect the protein content. **g** In vitro release profiles of RNPs and McM/RNPs (*n* = 6)

To synthesize McM/RNPs, the plasma membrane derived from monocyte was coated onto RNP cores by the coextrusion method. First, RNPs were obtained through the nanoprecipitation method. The hydrodynamic diameter of RNPs was 71 nm (Fig. 1a), and the zeta potential was − 27.8 mV by dynamic light scattering (DLS) (Fig. 1b). The average drug loading efficiency (LE) and drug encapsulating efficiency (EE) of RNPs were 13.15% and 59.16%, respectively (Fig. 1c). These results indicate that RNPs were successfully prepared by the nanoprecipitation method.

Next, monocyte membranes (McM) were extracted by disrupting monocytes and removing the intracellular content. Then, RNPs were mixed with fresh McM and coextruded through a 200 nm porous polycarbonate membrane to form McM/RNPs. The transmission electron microscopy (TEM) measurements revealed that, compared with uncoated RNPs, the diameter of McM/RNPs almost increased by 16 nm, which was attributed to the thickness of McM is about 8 nm. Additionally, the zeta potential of McM/RNPs (− 38.1 mV) was comparable to that of the original McM (− 50.8 mV) but much lower than that of unmodified RNPs (− 27.8 mV) (Fig. 1b). The TEM image results showed that McM had the structure of cell membrane (Additional file 1: Fig. S1) and McM/RNPs displayed a characteristic “core–shell” structure, which was consistent with unilamellar membrane coating around the polymeric core (Fig. 1d). Furthermore, we analyzed the protein content of monocytes, McM, McM/RNPs and RNPs by sodium dodecyl sulfate polyacrylamide gel electrophoresis (SDS-PAGE). The results showed that McM-camouflaged nanoparticles preserved the majority of the associated membrane proteins from monocytes (Fig. 1f). To analyze specific protein markers on McM/RNPs, Western blotting analysis was performed. The specific protein signals of integrin α4, integrin β1 and CD47 were observed in monocyte lysates, McM and McM/RNPs (Fig. 1e), which indicates that the membrane-modified nanoparticles maintained the characteristics of monocytes. Overall, a series of quality assurance specifications for the production of McM/RNPs was established to ensure physicochemical and biological reproducibility of the nanoparticles.

The release kinetics of RAP from RAPNPs and McM/RNPs were investigated in 1× PBS, pH 7.4, in vitro. At 5 days, 20.74% and 18.90% of RAP were released from RNPs and McM/RNPs, respectively. At 9 days, 24.82% and 20.51% of RAP were released from RNPs and McM/RNPs, respectively. Compared to RNPs, McM/RNPs showed a slightly slower RAP release profiling (Fig. 1g). In general, the steady and long-term RAP release behavior of McM/RNPs indicates their potential to be used for sustained drug release.

### Immune-escape function of McM/RNPs in vitro

**Fig. 2:**
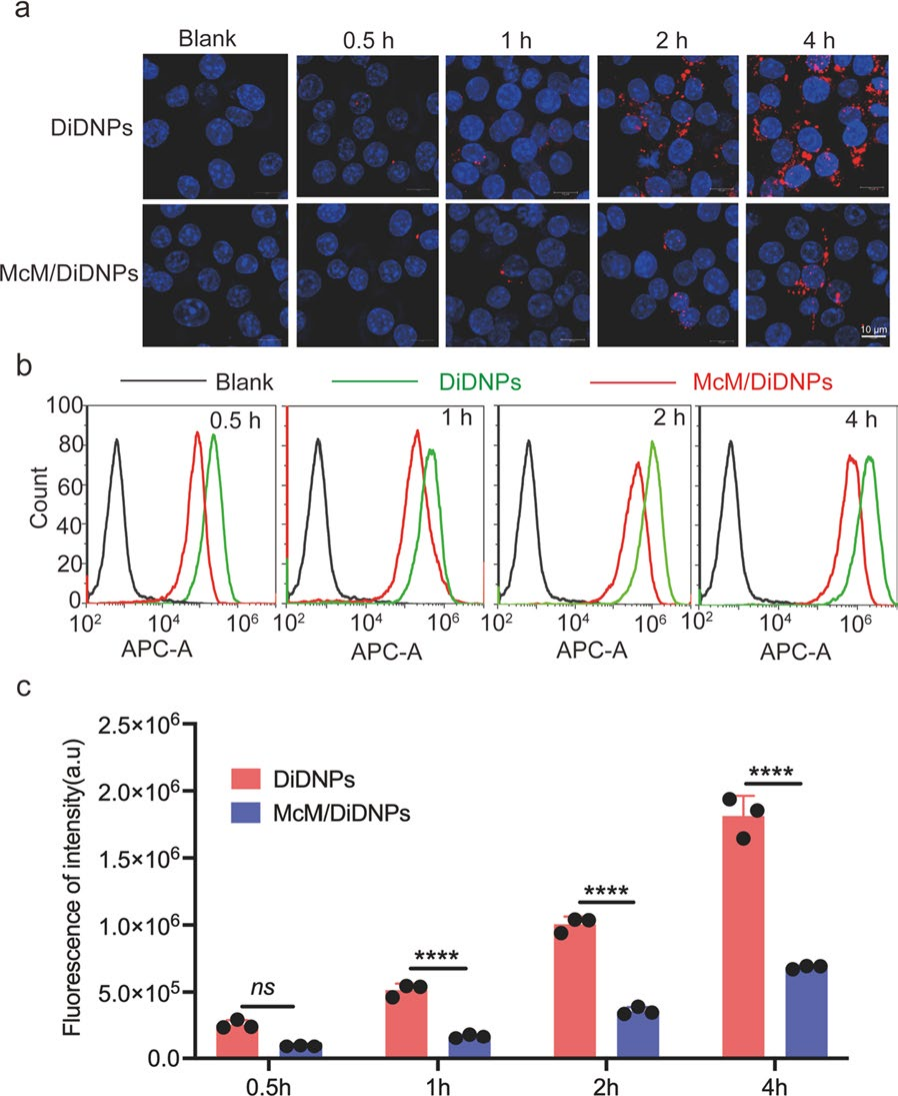
Cellular uptake nanoparticles by macrophages. **a** Confocal laser microscope images of DiDNPs and McM/DiDNPs internalized by macrophages (scale bar = 10 μm). **b** Cellular uptake of DiDNPs and McM/DiDNPs in macrophages by flow cytometry at different time points. **c** Quantification of the cellular uptake of DiDNPs and McM/DiDNPs in macrophages at different times points (*n* = 3). ****, *p* < 0.0001. *ns* no significance

Nanoparticles can be eliminated by the mononuclear phagocyte system in organisms [18]. To study the uptake of McM-coated nanoparticles by macrophages in vitro, we incubated DiDNPs and McM/DiDNPs with RAW264.7 cells and observed them with confocal laser scanning microscopy (CLSM). As shown in Fig. 2a, both DiDNPs and McM/DiDNPs were engulfed by macrophages in a time-dependent manner. However, the relative red fluorescent intensity in the DiDNPs group was stronger than that in McM/DiDNPs cells. Next, nanoparticles phagocytosed by macrophages were detected by flow cytometry analysis and the result was consistent with that from CLSM (Fig. 2b). After incubation for 0.5, 1, 2 and 4 h; the fluorescence intensity of DiDNPs was approximately 2.73, 3.11, 2.83 and 2.65 times higher than that of McM/DiDNPs, respectively (Fig. 2c). In addition, to confirm if nanoparticles engulfed by the macrophages, the lysosomes of macrophages were stained by Lyso-Tracker Green after incubating with nanoparticles. As showed in Additional file 1: Fig. S2, the nanoparticles almost all co-located with lysosomes in both DiDNPs and McM/DiDNPs groups, which indicated that the nanoparticles are engulfed by the macrophages. Overall, the above results show that McM-coated nanoparticles can significantly inhibit the phagocytosis of macrophages, which indicates that McM/RNPs have the potential to prolong the blood circulation time by reducing undesirable clearance. This result is also consistent with the previous reports that nanoparticles coated with a cell membrane can inhibit the phagocytosis of macrophages [19–21].

### McM/RNPs inhibit the adhesion of monocytes on inflammatory endothelium

**Fig. 3:**
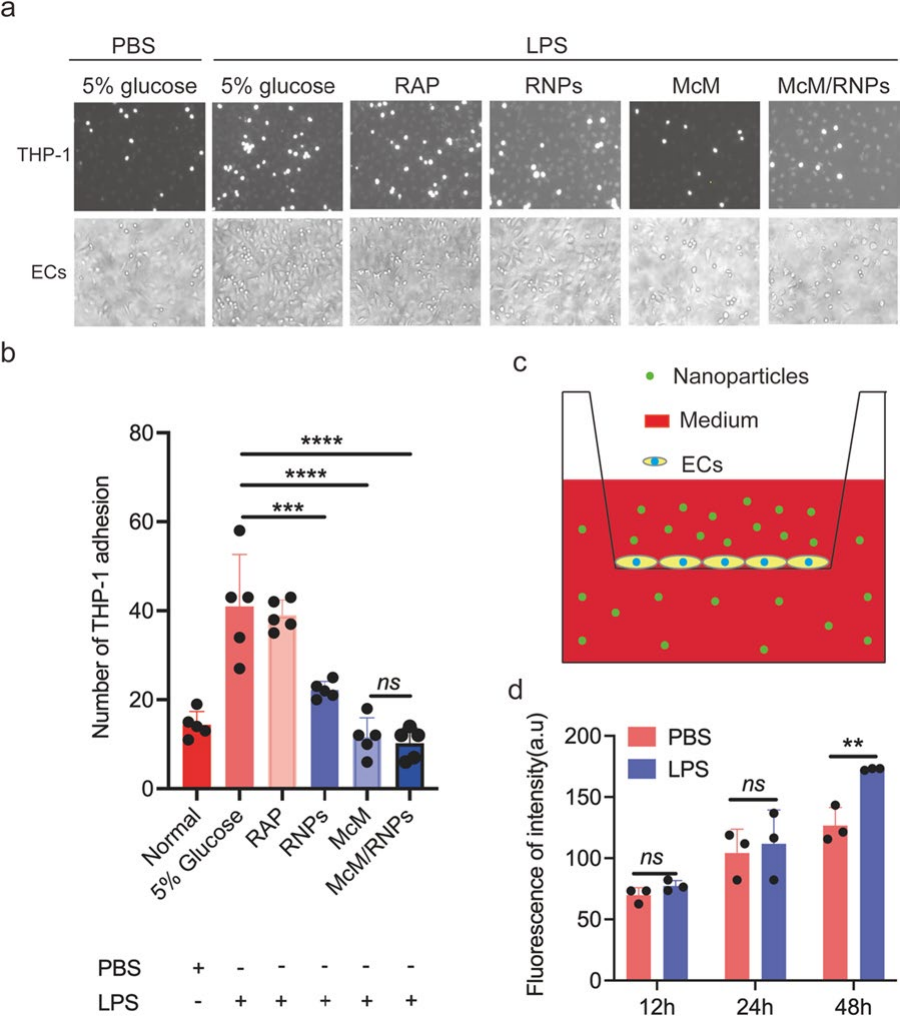
The adhesion of monocytes on inflammatory endothelial cells and the transmigration capability of McM-coated nanoparticles. **a** Images of monocyte adhesion on HUVECs after different treatments. **b** Quantification of monocyte adhesion (*n* = 5). **c** Schematic illustration of the Transwell model to evaluate the penetration capability of McM-coated nanoparticles across the inflamed endothelium. **d** Quantification of the fluorescence intensity of McM/DiDNPs in the bottom chamber at different time points. **, *p* < 0.01. ***, *p* < 0.001. ****, *p* < 0.0001. *ns*, no significance

There is substantial evidence that I/R injury was associated with inflammation [22, 23] and endothelium-monocyte adhesion is a critical road for inflammatory cell infiltration to the injury site of I/R [11]. Therefore, blocking the adhesion of monocytes to the inflammatory endothelium may be beneficial to alleviate I/R injury. To test whether McM/RNPs can resist inflammatory endothelial-monocyte adhesion, we first utilized lipopolysaccharide (LPS) to induce human umbilical vein endothelial cell (HUVEC) inflammation in vitro. Both vascular cell adhesion molecule-1 (VCAM-1) and intercellular cell adhesion molecule-1 (ICAM-1) were upregulated after HUVECs were stimulated with LPS for 12 h (Additional file 1: Fig. S3). Then, monocytes were incubated with inflammatory HUVECs which were pretreated with RAP, RNPs, McM, or McM/RNPs. The results showed that RNP may have the non-specific block function on ECs to inhibit monocyte infiltration, McM and McM/RNPs significantly inhibited the adhesion of THP-1 cells to inflammatory HUVECs (Fig. 3a, b).

In addition, we investigated the transmigration capability of McM coated nanoparticles in HUVECs by using in vitro Transwell migration assays [24, 25]. The Transwell model was established by incubating monolayer HUVECs on the top chamber (Fig. 3c). After incubation with or without LPS in the bottom chamber at 37 °C for 24 h, McM/DiDNPs were added to the upper chamber of the model, followed by testing of the fluorescence intensity of the culture medium in the bottom chamber at different time points. As shown in Fig. 3d, the transmigration of McM-coated nanoparticles could be promoted with the addition of LPS, especially after incubation for 48 h. Together, these results demonstrate that McM-coated nanoparticles have the capability to retard inflammatory cell binding and penetration to inflamed endothelium, which are of important significance for subsequent in vivo I/R injury treatment.

### In vivo long-term circulation and targeting of McM/RNPs to the I/R injury areas

To assess whether McM/DiDNPs inherited a long circulation lifetime from the natural McM, we studied the pharmacokinetics in vivo in a rat model. After intravenous injection via the tail vein, the residual content of the nanomedicine was evaluated by measuring the relative signal intensity of the collected blood at certain time interval using fluorescence microplate reader. The results showed that on the first day, the residual fluorescence of DiDNPs in the blood was about 45.41%, while that of McM/DiDNPs were 68.85%. At the 6th day, there was almost no DiDNPs remaining in the blood (about 0.22%), while McM/DiDNPs was 5.63% (Additional file 1: Fig. S4). Therefore, the McM/DiDNPs exhibited superior blood retention, suggesting that the immuno-suppressive surface makeup of the McM is able to efficiently prolong the blood circulation time.

**Fig. 4:**
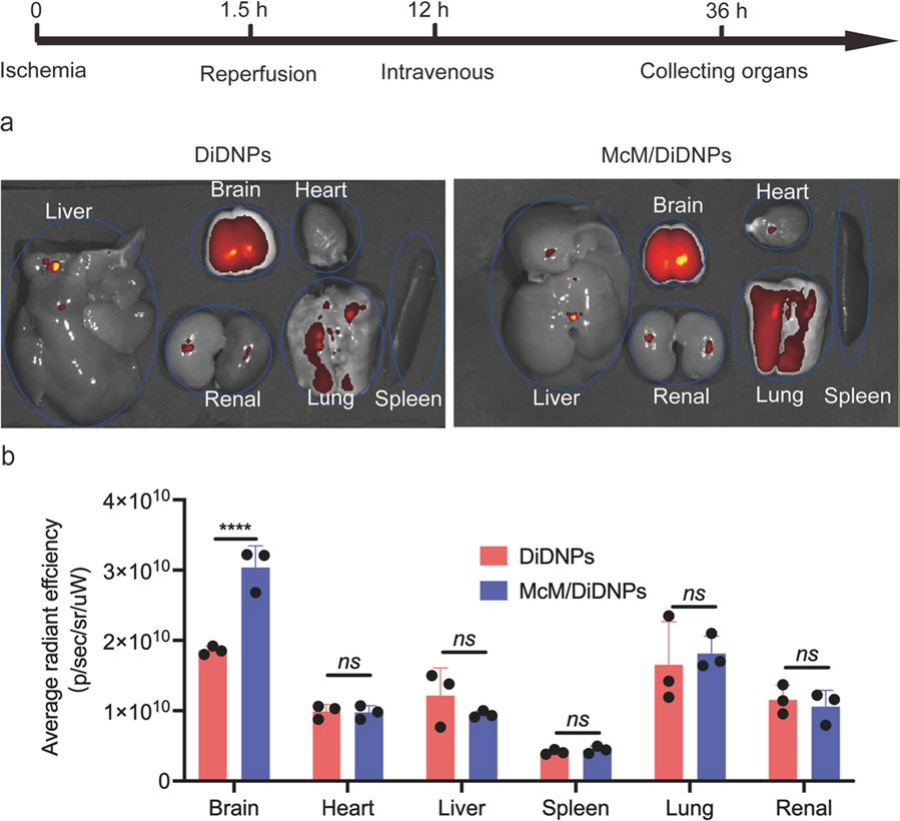
**a** Ex vivo fluorescent images of DiDNPs and McM/DiDNPs in the major organs at 24 h after *i.v.* injection. **b** Quantitative fluorescence intensity of DiDNPs and McM/DiDNPs in major organs (*n* = 3). ****, *p* < 0.0001. *ns* no significance

The in vivo targeting ability of McM/RNPs to the I/R injury areas was assessed after construction of a rat model of transient middle cerebral artery occlusion (tMCAO). To investigate McM-coated NPs accumulation in I/R injury areas, equivalent DiDNPs and McM/DiDNPs were *i.v.* injected into the tMCAO rats. After 24 h, rats were sacrificed, and the main organs were harvested for ex vivo imaging. The fluorescent intensity observed in the brain, suggesting that more therapeutic agents accumulated in the brain sections via McM camouflage (Fig. 4 and Additional file 1: Fig. S5). In addition, the fluorescence signal also showed that both DiDNPs and McM/DiDNPs were mainly distributed in the heart, liver, kidney and lung, including the brain. However, the fluorescence signal of the McM/DiDNPs group was slightly lower than that of the DiDNPs group in the liver and kidney. This result indicates that McM coating can reduce the accumulation of nanoparticles at major organs in vivo, which could reduce the side effects and nonspecific toxicity of McM-coated nanoparticles [17, 19–21].

### In vivo anti-ischemic stroke efficacy

**Fig. 5:**
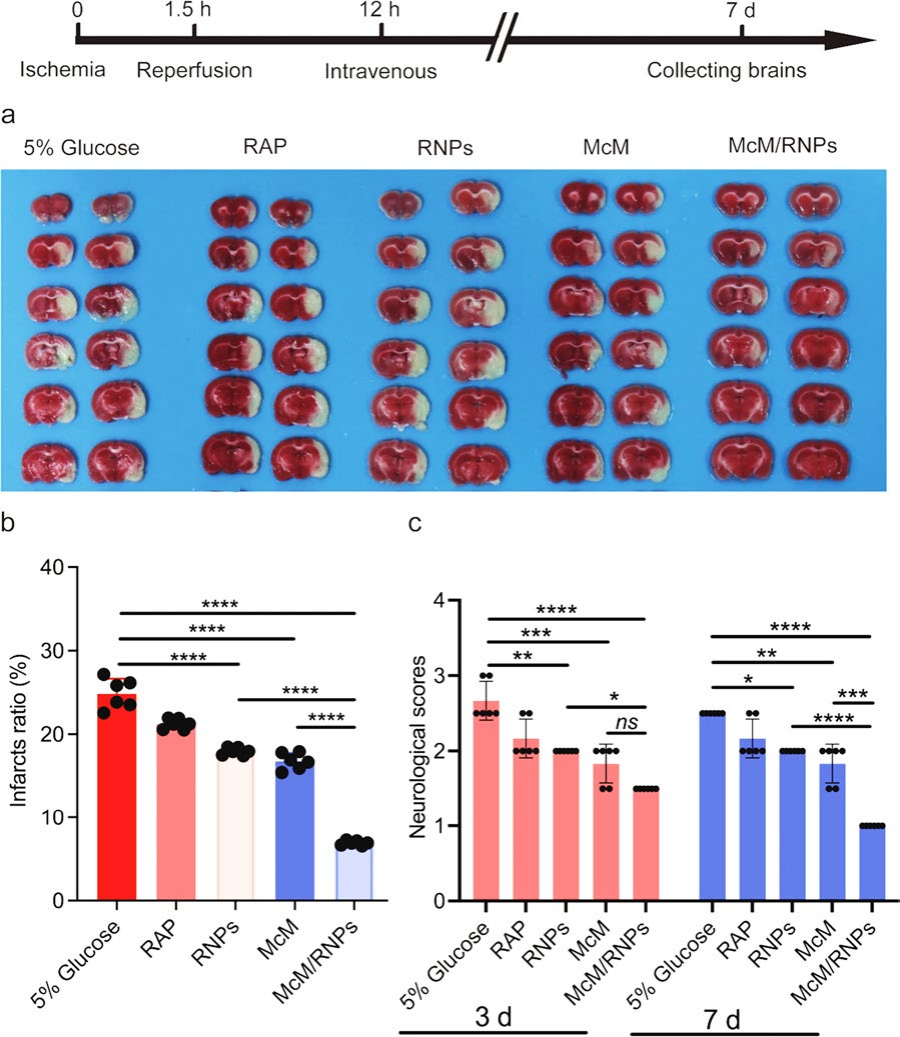
I/R treatment with different nanoparticles 7 days after tMCAO. **a** Representative TTC staining images of coronal sections (*n* = 6). **b** Quantified infarct ratio (*n* = 6) and **c** neurological scores (*n* = 6). *p < 0.05. **p < 0.01. ***p < 0.001. ****p < 0.0001

To substantiate the therapeutic potential of McM/DiDNPs, the in vivo anti-ischemic stroke effect of McM/RNPs was investigated in tMCAO rat models. Rat models were randomly divided into five groups and injected with 5% glucose, free RAP, RNPs, McM, and McM/RNPs 12 h after reperfusion. The infarct size was evaluated 7 days after ischemia–reperfusion. As shown in Fig. 5a–c, an obvious brain infarct was observed in the tMCAO rat group. When rats were treated with McM/RNPs, the brain infarct (right side of the image) was significantly ameliorated compared with that of the tMCAO group. Quantification of the infarcted brain using ImageJ showed that McM/RNPs decreased the infarct volume to 6.92% compared to 24.84% when the rat was treated with 5% glucose (Fig. 5b). Furthermore, rat neurological deficit scores were recorded, and treatment with McM/RNPs significantly ameliorated the neurological deficit induced by I/R compared to free RAP, RNPs and McM (Fig. 5c).

**Fig. 6:**
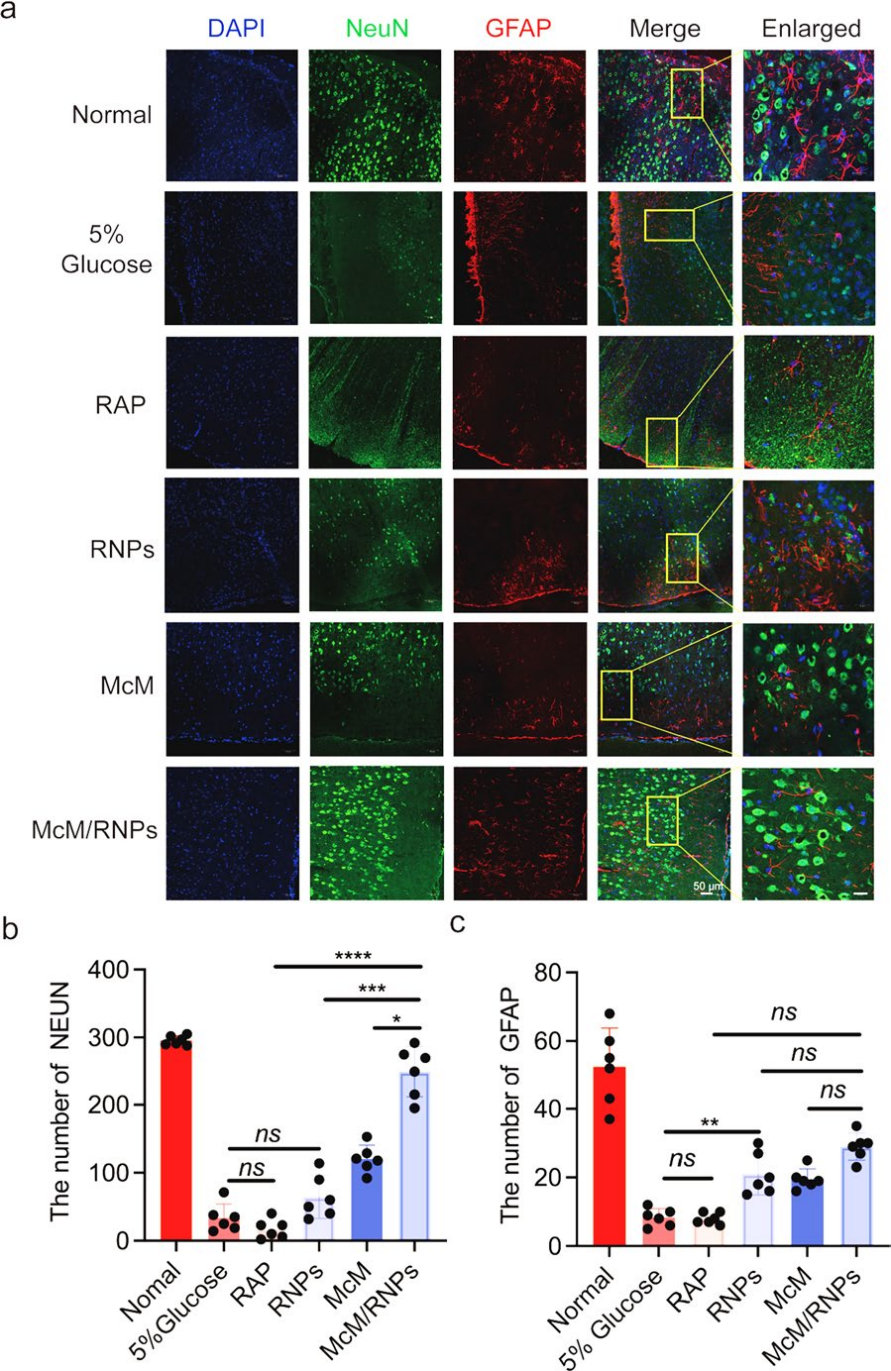
Representative immunofluorescent staining of NeuN and GFAP at 7 days after tMCAO treatment. **a** Immunofluorescence staining of NeuN and GFAP in the injury side after different treatments in tMCAO rats (scale bar = 50 μm). **b** Statistical analysis of NeuN in the injury side (*n* = 6). **c** Statistical analysis of GFAP in ipsilateral zone (*n* = 6). *p < 0.05. **p < 0.01. ***p < 0.001. ****p < 0.0001. *ns*, no significance

Moreover, the neurons (NeuN) and astrocyte (GFAP) in infarcted areas were examined through immunofluorescent staining [26]. As shown in Fig. 6, neurons and astrocytes were higher in the McM/RNPs treatment group compared with the free RAP, RNPs, and McM groups, and the number of neurons was almost the same as that of the normal group. These results indicate that McM/RNPs are an effective strategy for reperfusion-induced injury in ischemic stroke.

### McM/RNPs inhibit microglia proliferation and inflammation damage during acute cerebral ischemia

**Fig. 7:**
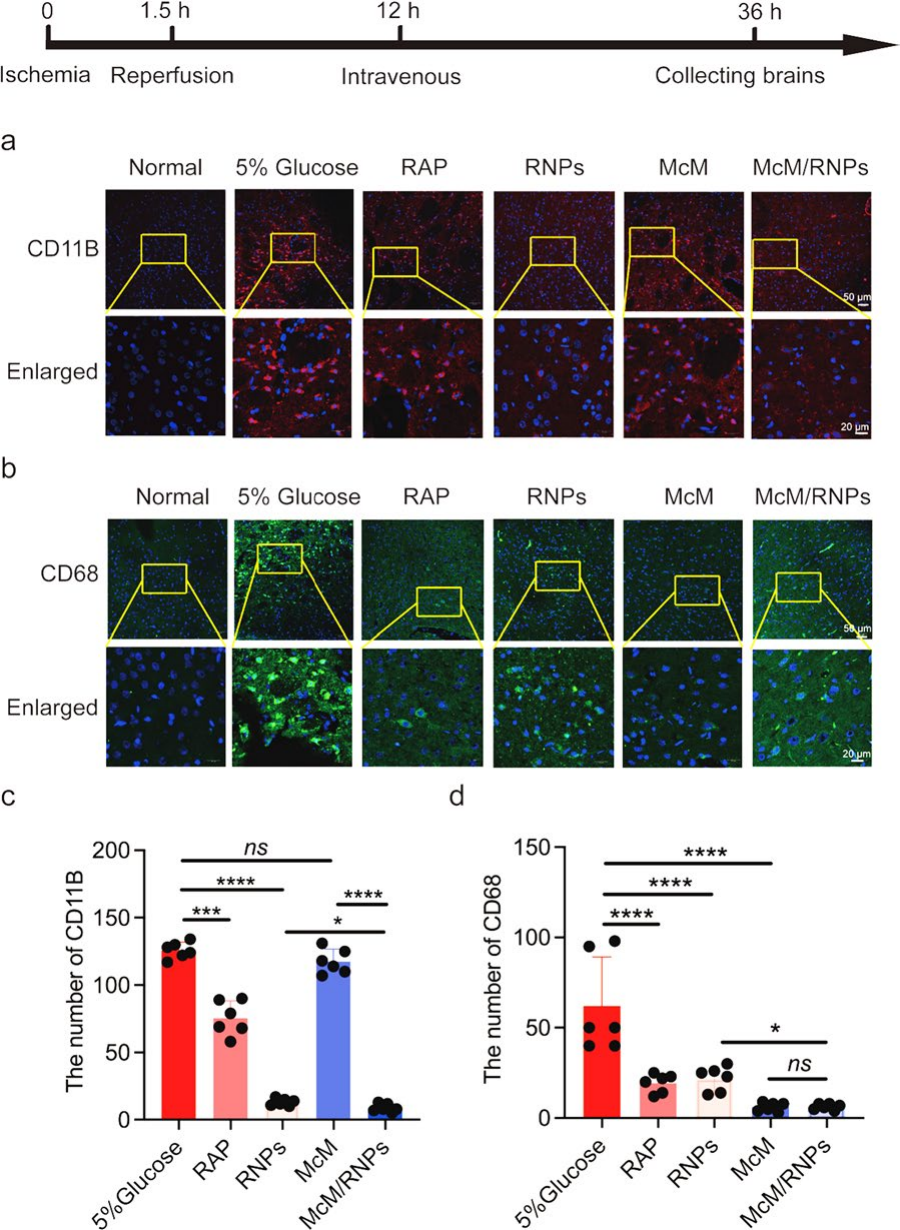
Representative inflammatory immunofluorescence staining of CD11B and CD68 at 1 day after tMCAO treatment. **a** Immunofluorescence staining of CD11B (scale bar = 50 μm. Enlarged, scale bar = 20 μm). **b** Immunofluorescence staining of CD68 (scale bar = 50 μm. Enlarged, scale bar = 20 μm). **c** Statistical analysis of the number of microglia in ischemic zone *(n* = 6). **d** Statistical analysis of the number macrophages in the ischemic zone (*n* = 6). *, *p* < 0.05. ***, *p* < 0.001. ****, *p* < 0.0001. *ns*, no significance

After brain I/R injury, a significant poststroke inflammatory response in the brain is defined by the activation and proliferation of microglia, which can release inflammatory and neurotoxic factors to promote the development of neurodegenerative diseases [27–29]. Immunofluorescence analyses for CD11B (a microglia marker; Fig. 7a, c) showed that the number of microglia dramatically decreased in infarcted areas, particularly in the RNPs and McM/RNPs treated groups. The results indicate that rapamycin-loaded nanoparticles can inhibit the proliferation of microglia in situ in the rat brain [26, 30]. Moreover, in addition to the activation and proliferation of microglia, the recruitment of macrophages from the blood is enhanced in I/R injury areas. The immunofluorescence images of CD68 (macrophages marker) showed that the number of macrophages in both the McM and McM/RNPs groups were significantly decreased after 1 day of treatment (Fig. 7b, d).

The above results demonstrate that McM/RNPs can reduce the inflammation (Fig. 7) and increase the repair of reperfusion-induce injury in ischemic stroke (Fig. 5). However, it seems that RNPs also have a strong inflammation inhibition with less injury repair than that of McM/RNPs. We speculate the main reasons are that: (1) RNPs are easy to penetrate to the injury site because it can directly cross the BBB and a lot of McM/RNPs may bind to the inflammatory endothelium in the first day; (2) RNPs may release RAP relatively fast in vivo (Fig. 1g), so it is better to control the inflammation at the first day, but the faster release of RAP may inhibit the neuron proliferation. (3) RNPs may have the non-specific block function on ECs to inhibit monocyte infiltration.

### In vivo biosafety assessment

**Fig. 8:**
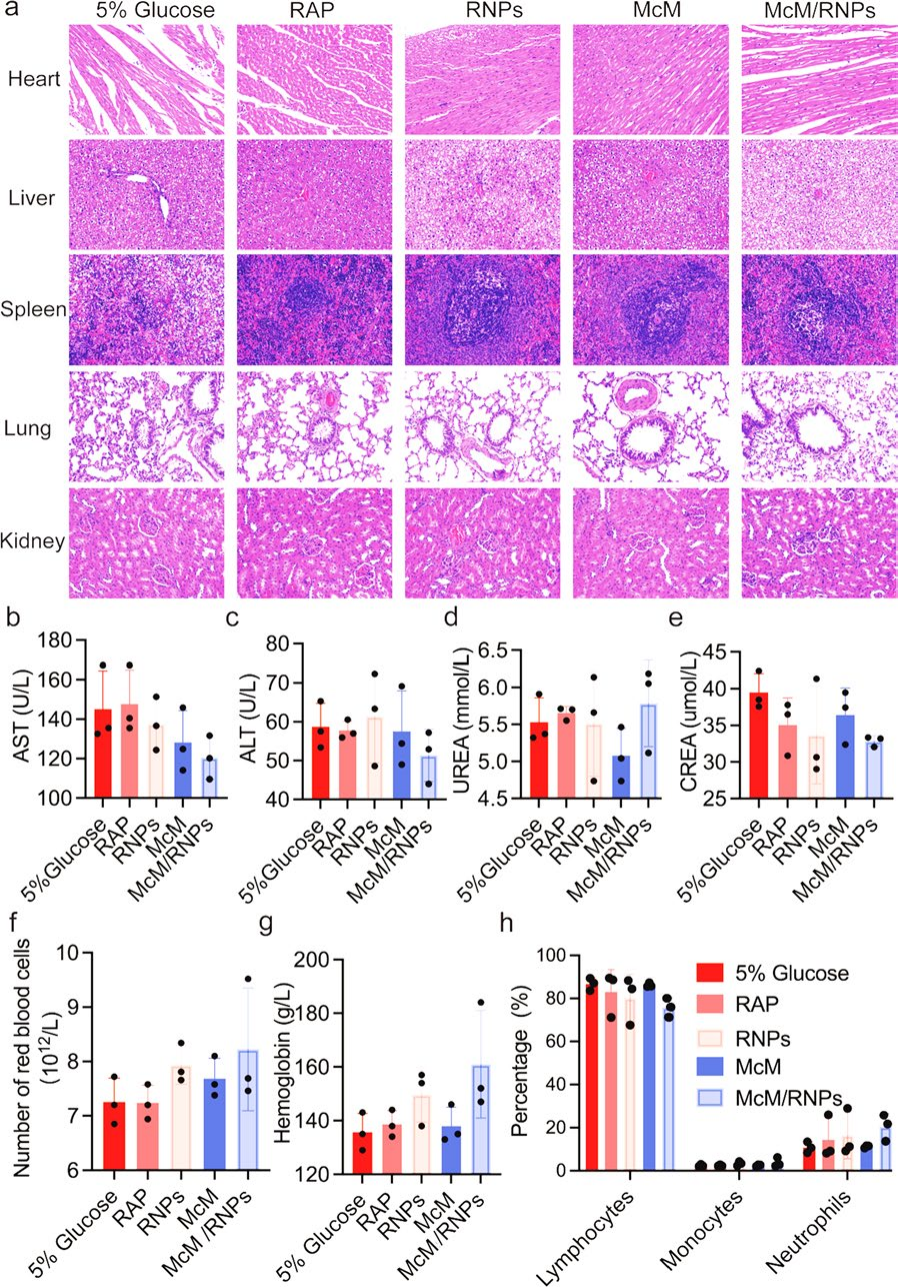
Preliminary safety evaluation. **a** H&E staining images of main organs from rats after different treatments. All micrographs were acquired at ×200 magnification. **b**–**e** Biochemical markers relevant to hepatic and kidney function (*n* = 3). **f** The percentage of inflammatory cells in the blood (*n* = 3). **g**, **h** Typical hematological parameters (*n* = 3)

To further assess biosafety, potential side effects were investigated after treatment. Hematoxylin–eosin (H&E) staining of the major organs showed no significant changes after different treatments (Fig. 8a). To further confirm treatment biocompatibility, hepatic and renal-related serum chemistries that reflect liver and kidney function were tested. As shown in Fig. 8b–e, the aspartate aminotransferase (AST), alanine aminotransferase (ALT), urea nitrogen (UREA) and creatinine (CREA) levels in the various groups were normal, which revealed that the functions of the liver and kidney were not impaired after treatment. Complete blood count implied that red blood cells and hemoglobin displayed no significant variations (Fig. 8f, g). Specifically, the counts of immune cells, including lymphocytes, monocytes, and neutrophils, in the blood of the nanoparticles-treated rats were similar to those in the 5% glucose group (Fig. 8h). In conclusion, McM/RNPs have no obvious side effects or immunotoxicity after treatment, making McM/RNPs a safe potential candidate for I/R injury treatment.

## Conclusion

Here, we fabricated a monocyte membrane-functionalized drug delivery system (McM/RNPs) to achieve synergistic immunochemotherapy therapy of ischemic stroke. In vitro evaluation showed that McM/RNPs can inhibit the uptake of macrophages, retard inflammatory cell binding to inflammatory HUVECs, and penetrate across the inflamed endothelium in a cell-based model. Furthermore, an ex vivo fluorescent imaging study showed that McM/RNPs exhibited improved active homing to I/R injury areas. Importantly, McM/RNPs can also greatly ameliorate neuroscores and infarct volume in response to surgical MCAO injury. Therefore, McM/RNPs may be utilized as a potential formulation strategy to enhance the treatment of ischemic stroke in the clinic.

## Materials and methods

### Drugs and materials

Poly (lactic-co-glycolic) acid (PLGA, MW 900000, 50:50) and RAP were purchased from Chengdu Good Alumni Biotechnology Co., Ltd. (Sichuan, China). 1,1ʹ-Dioctadecyl-3,3,3ʹ,3ʹ-tetramethylinddodicarbocyanine perchlorate (DiD) was purchased from Biotium Inc. (Fremont,US). Opti-mum cutting temperature compound (OCT), 2,3,5-triphenyltetrazolium chloride (TTC) and 4,6-diamidino-2-phenylindole (DAPI) were purchased from Beijing Solarbio Science & Technology Co., Ltd. (Beijing, China). An enhanced BCA protein assay kit, membrane and cytosol protein extraction kit and PMSF were purchased from Beyotime Institute of Biotechnology (Jiangsu, China). The rat IL-4 ELISA kit and rat IL-10 ELISA kit were supplied by MultiScience (Lianke) Biotech Co., Ltd. (Zhejiang, China). Polycarbonate porous membranes were supplied by Whatman International Ltd. (Maidstone, Kent, UK). Ultrapure water with a resistivity of 18.2 MΩ cm was used throughout the experiments.

### Preparation of the monocyte membranes

Monocyte membranes derived from RAW264.7 cells were extracted as previously described with slight modification [19, 31]. The cells were cultured in DMEM (Gibco) with 10% FBS and 1% penicillin–streptomycin (Gibco). Monocyte membranes were obtained using a Membrane and Cytosol Protein Extraction Kit. Three bottles of cells were collected and washed with PBS three times (2500 rpm for 5 min each) [32]. Then, 990 mL membrane protein extraction reagent A and 10 mL of PMSF (Beyotime) were added to an ice bath for 15 min. After that, the mixture was first frozen at − 80 ℃ and thawed at room temperature, and the cells were subjected to 3–5 freeze–thaw cycles [31, 33]. The obtained mixture was centrifuged at 1500 rpm (4 ℃, 10 min) to remove cytoplasm and various organelles. Next, the cell membranes were obtained at 12,000 rpm (4 ℃, 30 min) [16]. An enhanced BCA protein assay kit was used to analyze the total protein content in the obtained monocyte membranes. Those membranes (named McM vesicles) were stored at − 80 ℃ for later use.

### Fabrication of Rapamycin-loaded PLGA nanoparticles (RNPs)

PLGA and rapamycin (RAP) were dissolved in N, N-Dimethylformamide (DMF), and 40 mg/L and 10 mg/L solutions were generated, respectively. RNPs were obtained by the nanoprecipitation method as previously described, with a minor modification [33, 34]. Then, 250 mL of PLGA and 200 mL of RAP were added in 550 mL of DMF (total 1 mL mixture). The mixture was added dropwise to 3 mL ultrapure water with gentle stirring, and then transferred to a dialysis bag (molecular weight cutoff (MWCO) of 3500 Da) to remove free RAP and DMF. The RNPs solution was stored at 4 ℃. To obtain fluorescently labeled NPs, PLGA was mixed together with 1,1ʹ-dioctadecyl-3,3,3ʹ,3ʹ-tetramethylindodicarbocyanine (DiD) dissolved in N,N-Dimethylformamide (DMF) to form DiDNPs. For fluorescent imaging experiments, 0.1 wt% DiD (excitation = 644 nm, emission = 665 nm) was encapsulated into PLGA cores to form DiDNPs [35].

### Fabrication of McM camouflaged RNPs (McM/RNPs)

Membrane-coated RNPs were fabricated by a previously reported sonication [33] and coextrusion method [16]. Briefly, collected McM vesicles and RNPs were mixed at a membrane protein-to-polymer ratio of 1:1 (w/w). Next, the mixture was sonicated for 5 min in a FS30D bath sonicator with a frequency of 42 kHz and a power of 100 W. Then, McM/RNPs were obtained by using the Avestin mini extruder (Avestin, LF-1, Canada) extruded 10 times through a 200 nm polycarbonate porous membrane. McM and DiDNPs were mixed and McM/DiDNPs were formulated by the coextrusion method.

### Nanoparticle characterization

RNPs, McM vesicles, and McM/RNPs were measured for size and zeta potentials with dynamic light scattering (DLS) using a Malvern ZS 90 Zetasizer. The nanoparticle morphology was examined by transmission electron microscopy (TEM) at 200 kV (JEM-2100F, JEOL, Japan). TEM was performed by depositing the particles onto a 300-mesh glow-discharged carbon-coated grid (Beijing Zhongjingkeyi Technology Co., Ltd.) and negative staining with 1 wt% phosphotungstic acid.

### Drug loading and encapsulation efficiency of nanoparticles

Briefly, 1 mg of RAP was mixed with 10 mg PLGA copolymer at a weight ratio of 1:10 and dissolved in 1 mL of DMF. To calculate the loading efficiency of RAP, RNPs lyophilized power was dissolved in DMF, and the absorbance was measured using an ultraviolet spectrophotometer (DU730, Beckman Coulter) at 280 nm. According to the pre-established standard curve of RAP in DMF, the concentrations of the standard curve were 0, 2, 4, 8, 16 and 32 mg/mL. The drug loading efficiency (LE) and drug encapsulation efficiency (EE) were calculated by Eq. (1) and (2), respectively [16, 36].

1 $$\mathrm{LE }(\mathrm{\%})=\frac{Weight of RAP in lyophilized powder }{Weight of PLGA+Weight of RAP in lyophilized powder} \times 100\mathrm{\%}$$

2 $$\mathrm{EE }(\mathrm{\%})=\frac{Weight of RAP in lyophilized power}{Weight of added RAP} \times 100\mathrm{\%}$$

### SDS-PAGE gel and western blotting

Samples containing monocytes, McM, and McM/RNPs were denatured and loaded into a 10% SDS-polyvinylidene gel. The membrane proteins of the McM vesicles and McM/RNPs were extracted by Cell lysis buffer for Western blotting (Beyotime Institute of Biotechnology) and centrifuged at 12,000 rpm for 20 min at 4 ℃. Protein quantification was performed using a BCA Protein Assay kit (Beyotime Institute of Biotechnology). All the extracted proteins were run on a Bis–Tris 10-well mini-gel in running buffer using a Bio-Rad electrophoresis system at a constant of 80 V for 30 min and then at 120 V for 90 min. Next, the resulting polyacrylamide gel was stained with Coomassie blue for visualization.

Furthermore, the integrin a4, integrin β1, CD47, and b-actin contents in monocytes, McM, McM/RNPs, and RNPs were determined by Western blot analysis. Samples were subjected to 10% SDS-PAGE and transferred onto polyvinylidene fluoride (PVDF) membranes (Millipore, USA). The membranes were blocked with 5% nonfat milk in TBST buffer (10 mM Tris–HCl, 100 mM NaCl, pH 7.4, 0.1% Tween-20) at 25 ℃ for 1 h. Then, the membranes were incubated with primary antibodies: a4 (1:5000 dilution, CST), β1 (1:5000 dilution, CST), CD47 (1:1000 dilution, ABclonal) and β-actin (1:5000 dilution, GeneTex) at 4 ℃ overnight in the refrigerator. After the membranes were washed three times with TBST, they were further incubated with horseradish peroxidase-conjugated house anti-mouse secondary antibody (1:2000 dilution, Cell Signaling) or goat anti-rabbit secondary antibody (1:2000 dilution, Cell Signaling) at room temperature for 1 h. Protein bands were visualized by the enhanced chemiluminescence method using a ChemiDoc MP imaging system (Bio-Rad, USA).

### In vitro release of nanoparticles

The drug of RAP release from RNPs and McM/RNPs were studied using a dialysis method. Briefly, RAPNP and McM/RNP solutions (2 mg/mL, 3 mL each) were added to disposable dialysis bags (MWCO: 3500 Da, Thermo Scientific). The dialysis bags were then immersed in 12 mL of PBS solution (Release medium, pH 7.4) at room temperature. Three independent replicates were used for each sample. Two milliliters of release medium was collected for analysis at different time intervals (1 h, 2 h, 4 h, 8 h, 12 h, 1 day, 2 days, 3 days, 4 days, 5 days, 6 days, 7 days, 8 days and 9 days) and replaced with an equivalent volume of fresh PBS at room temperature. The cumulative amount of RAP released was quantified by a UV/Vis spectrophotometer (DU 730, Beckman Coulter) at 280 nm.

### Cellular uptake

To evaluate the effects of McM camouflaging on phagocytosis reduction by macrophages, experiments were performed using RAW264.7 macrophage cells. The internalization of DiDNPs and McM/DiDNPs by macrophages cells was evaluated by CLSM and FACS measurements. Briefly, RAW264.7 macrophage cells were seeded in 24-well plates at a density of 1 × 10^5^ cells per well in 500 μL of DMEM supplemented with 10% FBS and cultured for 12 h. Then, 150 μg of DiDNPs or McM/DiDNPs were added to each well. After coincubation for 0.5, 1, 2 and 4 h, the nuclei were stained with DAPI for CLSM imaging. Cells were collected for quantification by FACS analysis.

Quantification of NPs uptake in macrophages was measured by flow cytometry (BD, USA). Macrophages were seeded in 12-well plate with a density of 1 × 10^6^ cells/well. After 12 h incubation in 37 °C incubator containing 5% CO_2_, 100 μL (2.5 mg/mL) of DiDNPs or McM/DiDNPs solutions were added into each well, then the cells were incubated with the solutions for 0.5, 1, 2 and 4 h, respectively. After incubation, the cells were washed for three times with 1× PBS and digested with trypsin for collection. Then, the cells were washed with PBS twice before centrifuge at 1500 rpm for 5 min. Lastly, the cells were resuspended in 1 mL of 1× PBS. Three replicates were adopted for each group and 1 × 10^5^ cells were analyzed in each sample.To confirm that the nanoparticles were engulfed by the macrophages. RAW264.7 macrophage cells were seeded in 24-well plates at a density of 1 × 10^5^ cells per well in 500 μL of DMEM supplemented with 10% FBS and cultured for 12 h. After then, 150 μg of DiDNPs or McM/DiDNPs were added to each well. After incubate 4 h, the medium was changed to DMEM containing Lyso-Tracker Green. After coincubation, the nuclei were stained with Hoechst for 10 min. Then, the cells were washed twice with 1× PBS and imaged with a laser confocal microscope.

### In vitro Transwell assay

In the Transwell models, HUVECs were inoculated into the upper chamber with a diameter of 0.40 mm in a 24-well plate (1 × 10^5^ per well), while medium was added to the lower chamber and stimulated with lipopolysaccharides (LPS, 10 mg/mL). When the ECs became a monolayer, LPS was added for 12 h, and then the new medium was changed and cocultured with McM/DiDNPs at different times. The medium in the lower chamber was collected, and the fluorescence intensity was measured with an F-4700 fluorescence spectrophotometer (Japan).

### Monocyte adhesion assay

Human umbilical vascular endothelial cells (HUVECs) grown to confluency in 24-well plates were stimulated with LPS (10 mg/mL) for 12 h and washed with PBS. THP-1 cells (Cell Bank of Chinese Academy of Sciences) were labeled with Hoechst. Next, the labelled THP-1 cells were overlaid on the HUVECs and incubated for 2 h. After washing, the numbers of THP-1 cells adhering to the HUVECs monolayer in each well were examined by a fluorescence microscope (Olympus Corporation, Japan). Finally, the number of THP-1 cells was counted by ImageJ software.

### Animal experiments

All experiments were performed using male Sprague–Dawley rats (SD, 280–320 g), purchased from Hunan SJA Laboratory Animal Co. Ltd. (Hunan, China). Procedures that used animals were reviewed and approved by the Tumor Hospital of Chongqing University Ethics Committee and in accordance with the Guide for the Care and Use of Laboratory Animals (Institute for Laboratory Animal Research, National Research Council, Washington, DC: National Academy Press, 1996). The rats were housed under alternating 12 h light/dark conditions and allowed free access to water and food before all experiments. The rats were divided into 5 groups: 5% glucose, RAP, RNPs, McM, or McM/RNPs.

Transient middle cerebral artery occlusion (tMCAO) models were generated according to methods that were recently reported [37–39]. Each SD rat was anesthetized with 5% isoflurane (Aerrane, Baxter, Deerfield, IL) in a mixture of 70% nitrous oxide and 30% oxygen. Isoflurane was then maintained at 1.5%. During the procedures, the body temperature of the rats was monitored at 37.0 ± 0.5 ℃. Rats were placed in the supine position and the right common carotid artery (CCA), external carotid artery (ECA), and internal carotid artery (ICA) were carefully exposed and dissected from the surrounding tissue. Then, a small hole in the ECA was made using Vanes-style spring scissors. A silicon-coated monofilament (Supplied by Guangzhou Jialing Biotechnology Co., Ltd. Guangdong, China) inserted into the ICA via the CCA, 8–10 mm away from the bifurcation, blocked the starting point of the middle cerebral artery. Successful MCA occlusion was confirmed by a reduction in rCBF by over 80%. The occlusion lasted 90 min, and the monofilament was withdrawn to allow for reperfusion.

### Long-term circulation function of nanoparticles in vivo

DiDNPs and McM/DiDNPs were administered in rats via the tail vein at a nanoparticle dosage of 2 mg/kg. 1 mL of 0.09% physiological saline was added to anticoagulation tube. At different time intervals (1, 2, 3, 4, 5 and 6 days), the tail of the rats were pierced, then 20 μL of whole blood was taken and dissolved in an anticoagulation tubes. The fluorescence intensity of the remaining nanoparticles in the blood were measured by fluorescence microplate reader.

### Ex vivo imaging

Rats with ischemic stroke were used for brain targeting evaluation nanoparticles [40]. Briefly, DiDNPs and McM/DiDNPs were administered via the tail vein at a nanoparticle dosage of 2 mg/kg. After 24 h, the rats were euthanized and perfused with precooled 0.09% physiological saline to remove the blood and unbound nanoparticles. For ex vivo near-infrared fluorescence imaging, images were captured using a near-infrared fluorescence in vivo imaging system (IVIS Lumina III, Perkin Elmer, USA) and quantitatively analyzed using Living Image 5.0 software. The organs, including the brain, heart, liver, spleen, lung, and kidney were subsequently fixed and used for histological analysis in the main organs and the accumulation of DiDNPs or McM/DiDNPs in stroke lesions [41].

### 2,3,5-Triphenyltetrazolium chloride (TTC) staining

To identify the primary infarction, rats (*n* = 15) selected randomly from ischemia in the 5% glucose, RAP, RNPs, McM, and McM/RNPs groups (3 rats per group) were sacrificed 7 days after MCAO under deep anesthesia as above and 2 mm thick coronal brain sections were prepared and immersed in 2% TTC (Solarbio, China) in saline for 20 min at 37 °C and fixed for 30 min in 4% paraformaldehyde [42].

### Immunofluorescence staining

Immunofluorescence was performed as previously described [43–46]. Frozen brain Sects. (10 mm) were prepared using a cryostat (Leica, CM1900) according to standard procedures. Briefly, sections were preincubated with 0.5% Triton X-100 (v/v) and 5% BSA in PBS for 10 min, followed by blocking in 5% bovine serum albumin (BSA) for 1 h at room temperature. Then, sections were incubated overnight at 4 ℃ with primary antibodies diluted in QuickBlock™ Primary Antibody Dilution Buffer for Immunol Staining (Supplied by Beyotime). The following primary antibodies were used: mouse anti-VCAM-1 (1:50, Santa Cruz, CA, USA), mouse anti-ICAM-1 (1:50, Santa Cruz, CA, USA), mouse anti-NeuN (1:200, Millipore, Bedford, USA), rabbit anti-GFAP (1:200, Abclonal, Wuhan, China), mouse anti-rat CD11B (1:150, Millipore, Bedford, USA), and mouse anti-CD68 (1:100, Abcam, USA). After washing the unbound antibody three times, the cells were washed in PBST (0.05% Tween-20 in PBS). The sections were incubated with secondary antibodies for 1 h at room temperature. The secondary antibodies were Alexa Fluor® 555 Donkey polyclonal secondary antibody to rabbit IgG-H&L (1:500, Abcam, USA) or Alexa Fluor® 647 Donkey polyclonal secondary antibody to mouse IgG-H&L (1:500, Abcam, USA). The cells were washed three times with PBST and counterstained for nuclei with DAPI (Solarbio, Beijing, China). Antifade mounting medium (Beyotime, Jiangshu, China) was used, and fluorescent images were obtained using a confocal laser scanning microscope (TCS SP8, Leica, Germany).

### Neurological functional assessment

Rat behavioral testing and scoring were assessed by two people following the instructions adopted from Bederson [47, 48]. Neurological scores were determined 3 and 7 days after MCAO, the effective neurological score of each rat was the average of neurological score from the two observers. Bederson scores were used to evaluate global neurological function as previously described [49]. Briefly, the rats were suspended by the tail 20 cm above the floor. The animals were scored based on the symptoms of the rats: 0 points, rats behave normally; 1 point, rats cannot fully stretch their left front legs; 2 points, rats turn around into a circle; 3 points, rats fall down to the left side; and 4 points, rats cannot move by themselves, losing their consciousness.

### Complete blood count and clinical chemistry

Blood was collected in EDTA spray-coated tubes and immediately analyzed for hematological parameters by an automated hematology analyzer (Sysmex KX-21, Sysmex Co., Japan), such as red blood cells, hemoglobin (HGB), lymphocytes, monocytes and neutrophils. The plasma concentrations of aspartate aminotransferase (AST), alanine aminotransferase (ALT), blood urea nitrogen (UREA), and creatinine (CREA) were quantified by an automated analyzer platform (Roche Cobas C501, Roche Co., Switzerland).

### Statistical analysis

GraphPad Prism software (GraphPad, USA) was used for statistical analysis. Two-tailed Student’s t test was used for comparison between two groups. Comparisons among three or more groups were performed using one-way ANOVA. Statistical significance levels were set to * *p* < 0.05, ** *p* < 0.01, *** *p* < 0.001 and **** *p* < 0.0001, with all data displayed as the mean ± SD.

## Supplementary Information

**Additional file 1: Fig. S1.** Transmission electron microscopy image of McM (scale bar = 500 nm). **Fig. S2.** Confocal laser microscope images of Lysosomes (green) and nanoparticles (red) (scale bar = 20 μm). **Fig. S3.** Immunofluorescence of VCAM-1 and ICAM-1 in HUVECs treated with PBS and LPS for 12h (scar bar = 20 μm). **Fig. S4.** Relative fluorescence intensity of DiDNPs and McM/DiDNPs for pharmacokinetic studies in Rats (n = 6). **Fig. S5.** *Ex vivo* fluorescent images of DiDNPs and McM/DiDNPs in the major organs at 24 h after *i.v.* injection.

## Data Availability

All date generated or analyzed during this study are included in this published article.
