# Supplementary material for: Functionalized nanoparticles with monocyte membranes and rapamycin achieve synergistic chemoimmunotherapy for reperfusion-induced injury in ischemic stroke
Source: J Nanobiotechnology. 2021 Oct 21;19:331. doi: 10.1186/s12951-021-01067-0 (PMC8529766; doi:10.1186/s12951-021-01067-0)
Supplement: Supplementary file 1 — Additional file 1: Fig. S1. Transmission electron microscopy image of McM (scale bar = 500 nm). Fig. S2. Confocal laser microscope images of Lysosomes (green) and nanoparticles (red) (scale bar = 20 μm). Fig. S3. Immunofluorescence of VCAM-1 and ICAM-1 in HUVECs treated with PBS and LPS for 12h (scar bar = 20 μm). Fig. S4. Relative fluorescence intensity of DiDNPs and McM/DiDNPs for pharmacokinetic studies in Rats (n = 6). Fig. S5. Ex vivo fluorescent images of DiDNPs and McM/DiDNPs in the major organs at 24 h after i.v. injection. [file 12951_2021_1067_MOESM1_ESM.docx]

**Functionalized nanoparticles with monocyte membranes and rapamycin achieve synergistic chemoimmunotherapy for reperfusion-induced injury in ischemic stroke**

*Yanyun Wang^1^, Yi Wang^1^, Shuyu Li^1^, Yuliang Cui^1^, Xiping Liang^2^, Juanjuan Shan^3^, Wei Gu^1^, Juhui Qiu^1*^, Yiliang Li^4*^, Guixue Wang^1*^*

wanggx@cqu.edu.cn; jhqiu@cqu.edu.cn; liyiliang93@163.com

1. Key Laboratory for Biorheological Science and Technology of Ministry of Education, State and Local Joint Engineering Laboratory for Vascular Implants, Bioengineering College of Chongqing University, Chongqing, 400030, China.
2. Department of Hematology-Oncology, Chongqing Key Laboratory of Translational Research for Cancer Metastasis and Individualized Treatment, Chongqing University Cancer Hospital, Chongqing, 400030, China.
3. Center for Precision Medicine of Cancer, Chongqing Key Laboratory of Translational Research for Cancer Metastasis and Individualized Treatment, Chongqing University Cancer Hospital, Chongqing, China.
4. The Eighth Affiliated Hospital, Sun Yat-sen University, Shenzhen, Guangdong, 518033, China.


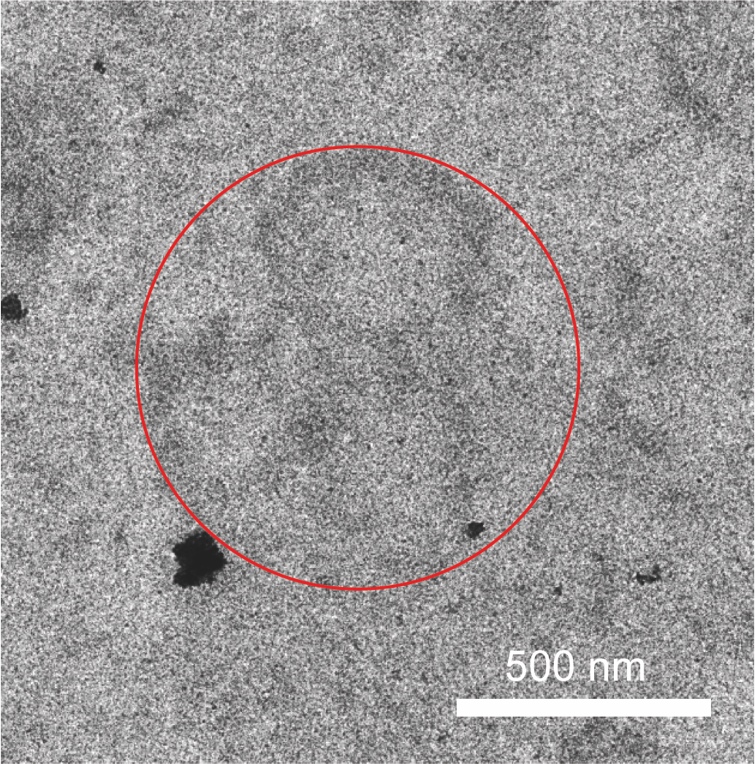


Supplementary Fig. 1. Transmission electron microscopy image of McM (scale bar = 500 nm).


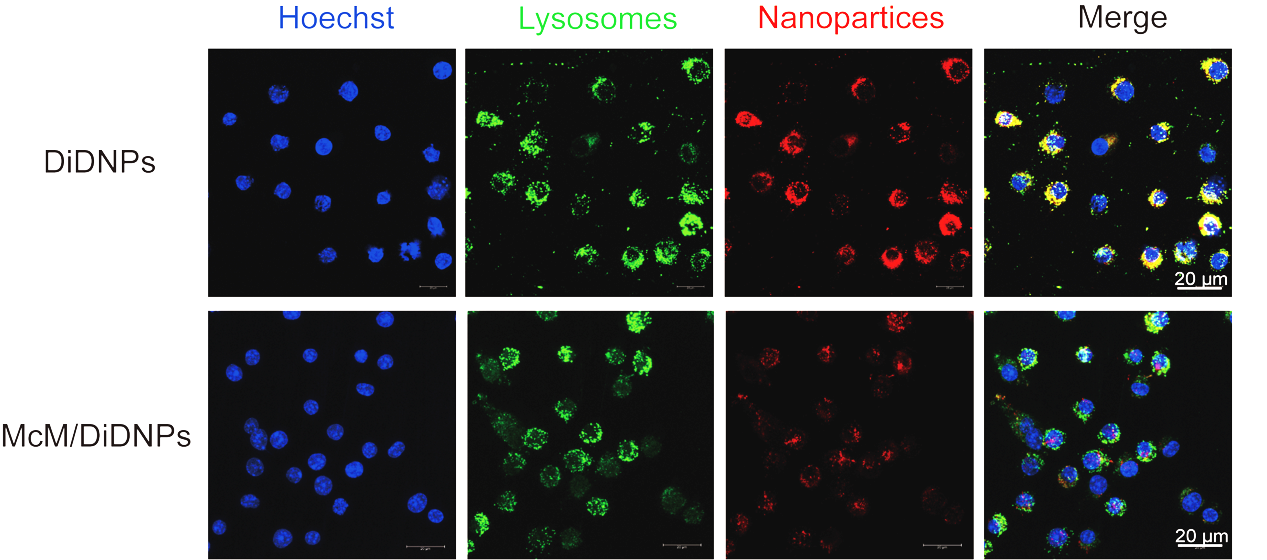


Supplementary Fig. 2. Confocal laser microscope images of Lysosomes (green) and nanoparticles (red) (scale bar = 20 μm)


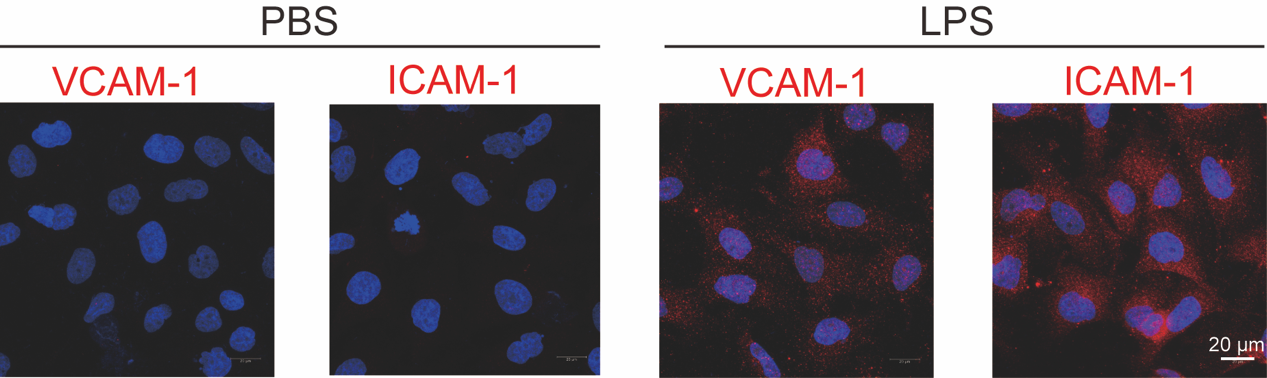


**Supplementary Fig.** 3. Immunofluorescence of VCAM-1 and ICAM-1 in HUVECs treated with PBS and LPS for 12h (scar bar = 20 μm).


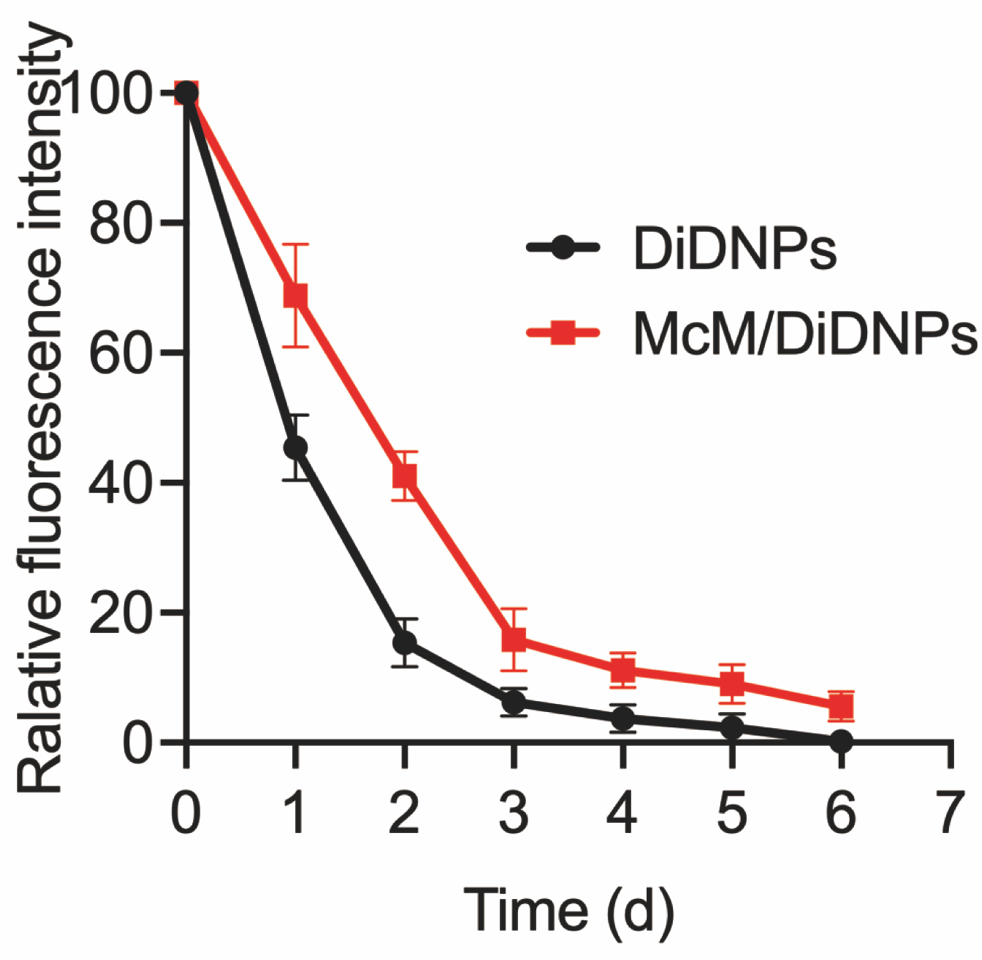


Supplementary Fig. 4. Relative fluorescence intensity of DiDNPs and McM/DiDNPs for pharmacokinetic studies in Rats (*n* = 6).


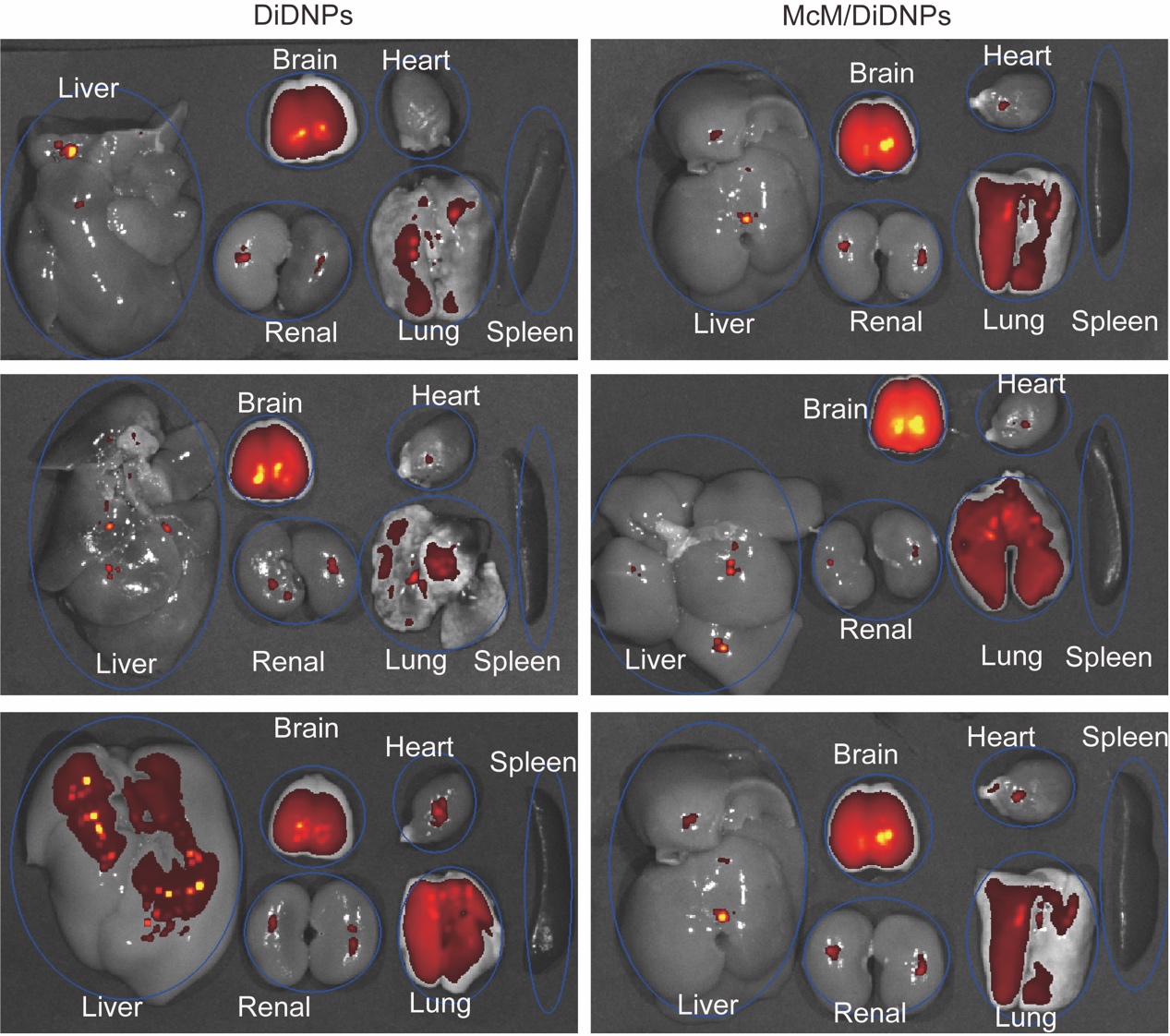


**Supplementary Fig.** 5. *Ex vivo* fluorescent images of DiDNPs and McM/DiDNPs in the major organs at 24 h after *i.v.* injection.
